# Surveillance and response systems for elimination of tropical diseases: summary of a thematic series in *Infectious Diseases of Poverty*

**DOI:** 10.1186/s40249-016-0144-7

**Published:** 2016-05-14

**Authors:** Xia Zhou, Peiling Yap, Marcel Tanner, Robert Bergquist, Jürg Utzinger, Xiao-Nong Zhou

**Affiliations:** Department of Parasitology, Medical College of Soochow University, No. 199 Renai Road, Suzhou, 215123 People’s Republic of China; National Institute of Parasitic Diseases, Chinese Center for Disease Control and Prevention, 207 Rui Jin Er Road, Shanghai, 200025 People’s Republic of China; Swiss Tropical and Public Health Institute, P.O. Box, CH-4002 Basel, Switzerland; University of Basel, P.O. Box, CH-4003 Basel, Switzerland; Ingerod 407, S-454 94 Brastad, Sweden; Key Laboratory on Parasite and Vector Biology, Ministry of Health, WHO Collaborating Centre for Topical Diseases, 207 Rui Jin Er Road, Shanghai, 200025 People’s Republic of China

**Keywords:** Infectious diseases, Tropical diseases, Health systems, Surveillance and response systems, Elimination, People’s Republic of China

## Abstract

The peer-reviewed journal *Infectious Diseases of Poverty* provides a new platform to engage with, and disseminate in an open-access format, science outside traditional disciplinary boundaries. The current piece reviews a thematic series on surveillance-response systems for elimination of tropical diseases. Overall, 22 contributions covering a broad array of diseases are featured – i.e. clonorchiasis, dengue, hepatitis, human immunodeficiency virus/acquired immune deficiency syndrome (HIV/AIDS), H7N9 avian influenza, lymphatic filariasis, malaria, Middle East respiratory syndrome (MERS), rabies, schistosomiasis and tuberculosis (TB). There are five scoping reviews, a commentary, a letter to the editor, an opinion piece and an editorial pertaining to the theme “Elimination of tropical disease through surveillance and response”. The remaining 13 articles are original contributions mainly covering (i) drug resistance; (ii) innovation and validation in the field of mathematical modelling; (iii) elimination of infectious diseases; and (iv) social media reports on disease outbreak notifications released by national health authorities. Analysis of the authors’ affiliations reveals that scientists from the People’s Republic of China (P.R. China) are prominently represented. Possible explanations include the fact that the 2012 and 2014 international conferences pertaining to surveillance-response mechanisms were both hosted by the National Institute of Parasitic Diseases (NIPD) in Shanghai, coupled with P.R. China’s growing importance with regard to the control of infectious diseases. Within 4 to 22 months of publication, three of the 22 contributions were viewed more than 10 000 times each. With sustained efforts focusing on relevant and strategic information towards control and elimination of infectious diseases, *Infectious Diseases of Poverty* has become a leading journal in the field of surveillance and response systems in infectious diseases and beyond.

**Electronic supplementary material:**

The online version of this article (doi:10.1186/s40249-016-0144-7) contains supplementary material, which is available to authorized users.

## Multilingual abstracts

Please see Additional file 1 for translations of the abstract into the six official working languages of the United Nations.

## Background

Tropical diseases are inextricably linked to populations living in resource-constrained settings [1, 2]. Infectious diseases of poverty, a collective term coined for infections known to be particularly prevalent amongst marginalized communities, is increasingly used to describe tropical diseases with special transmission routes, such as those that depend on a vector (e.g. malaria; vector is a mosquito) or an intermediate host (e.g. schistosomiasis; intermediate host is a snail) [3, 4].

The vision of the BioMed Central (BMC) open-access journal *Infectious Diseases of Poverty* is in line with the objectives put forth by the World Health Organization (WHO) in its 2012 Global Report [5]. The journal was launched in October 2012 to explore new avenues in research to deepen the understanding of the relationship between infectious diseases and poverty, to contribute to priority-setting and translation of key findings into policies and programmes to control and eliminate these diseases. The expanded “One World – One Health” concept, first proposed by the Wildlife Conservation Society and supported by the Food and Agriculture Organization (FAO), WHO, the World Bank and the United Nations Children’s Fund (UNICEF), recognises that the health of people, animals, and societies is intimately linked, and ultimately dependent, on the resilience of the world’s life-supporting ecosystems [6]. Based on this concept, *Infectious Diseases of Poverty* has published a series of original articles and empirical work with reference to analyses of disease burdens, their distributions and the research that is needed in this area. To raise global awareness of infectious diseases of poverty, the journal has not only published original research articles but also scoping reviews that highlight trans-disciplinary advances in the science undertaken. Several collections in different areas and on various topics have been published [3, 7].

The most recent collection, entitled “Surveillance and response to infectious diseases of poverty”, features 22 articles on topics ranging from tropical diseases to recent outbreaks of the H7N9 avian influenza A and the Middle East respiratory syndrome-coronavirus infection (MERS-CoV) with considerable international impact [8–10]. Eleven diseases are included, i.e. clonorchiasis, dengue, hepatitis, human immunodeficiency virus/acquired immune deficiency syndrome (HIV/AIDS), H7N9 avian influenza, lymphatic filariasis, malaria, MERS, rabies, schistosomiasis and tuberculosis (TB). The purpose of this overview is to summarise and systematise the research and shed new light on the plans for elimination of infectious diseases of poverty put forth in the current collection of the journal.

## Article collection

### The categories

This collection of articles published in *Infectious Diseases of Poverty* highlights surveillance-response systems that are geared towards the elimination of tropical diseases. It consists of 22 contributions; 13 original research articles, five scoping review, a commentary, a letter to the editor, an opinion piece and an editorial (Additional file 2).

The scoping reviews pertain to surveillance-response systems for elimination of tropical diseases, namely (i) surveillance-response systems as the key to eliminate tropical diseases [11]; (ii) surveillance of antimalarial drug resistance in the People’s Republic of China (P.R. China) [12]; (iii) challenges and needs to eliminate rabies in P.R. China [13]; (iv) a review focusing on TB at the wildlife-livestock-human interface in Zambia [14]; and (v) epidemiology and interactions of HIV-1 and *Schistosoma mansoni* in sub-Saharan Africa [15]. The commentary is linked to the rabies piece and discusses the science of elimination more broadly [16]. In a letter to the editor, research priorities in modelling the transmission risk of H7N9 avian influenza are discussed [17]. The opinion piece emphasises that clonorchiasis in P.R. China must be addressed and tackled in a timely manner [18]. The editorial pertains to the elimination of tropical diseases and highlights the importance of surveillance and response [8].

**Fig. 1:**
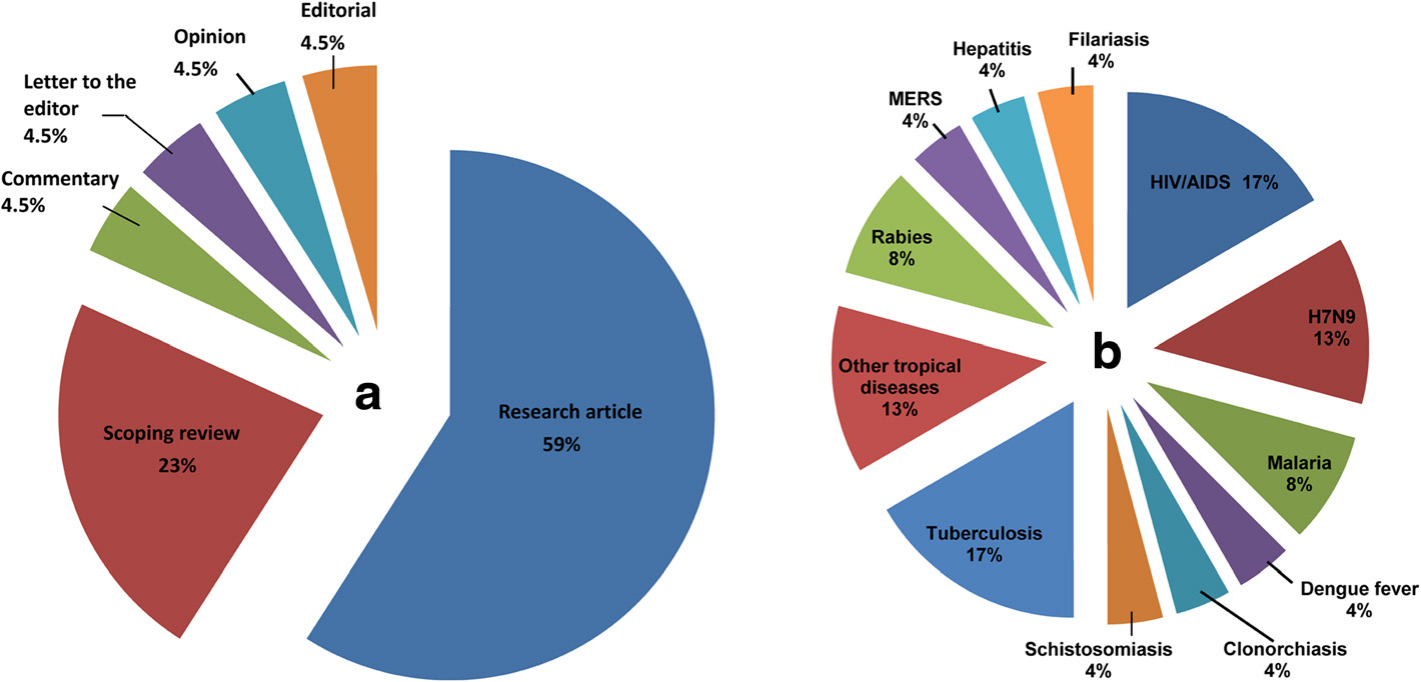
The categories of the 22 contributions (**a**) and the main topics discussed (**b**) in a collection of *Infectious Diseases of Poverty* pertaining to surveillance-response systems that might lead to elimination of tropical diseases

The 13 original research articles [10, 19–30] cover a broad array of topics along the entire value chain from innovation to application. The development and validation of mathematical models are discussed, social media communications arising from notifications of disease outbreaks are introduced and *in vitro* gene silencing (e.g. of independent phosphoglycerate mutase (iPGM) in *Brugia malayi* to identify the antifilarial drug targets) are featured. Importantly, one article pertains to health policy and planning with a specific focus on surveillance-response mechanisms (Fig. 1a).

### Diseases covered and author constellations

The 22 contributions focus on 11 different diseases, ranging from single diseases (i.e. clonorchiasis, dengue, hepatitis, HIV/AIDS, H7N9 avian influenza, lymphatic filariasis, malaria, MERS-CoV, rabies, schistosomiasis and TB) to co-morbidity with non-communicable diseases (e.g. hypertension and diabetes) or risk of infectious diseases among pregnant women. Several other infectious diseases (e.g. diarrhoea, measles and upper respiratory tract infections) are also addressed (Fig. 1b).

**Fig. 2:**
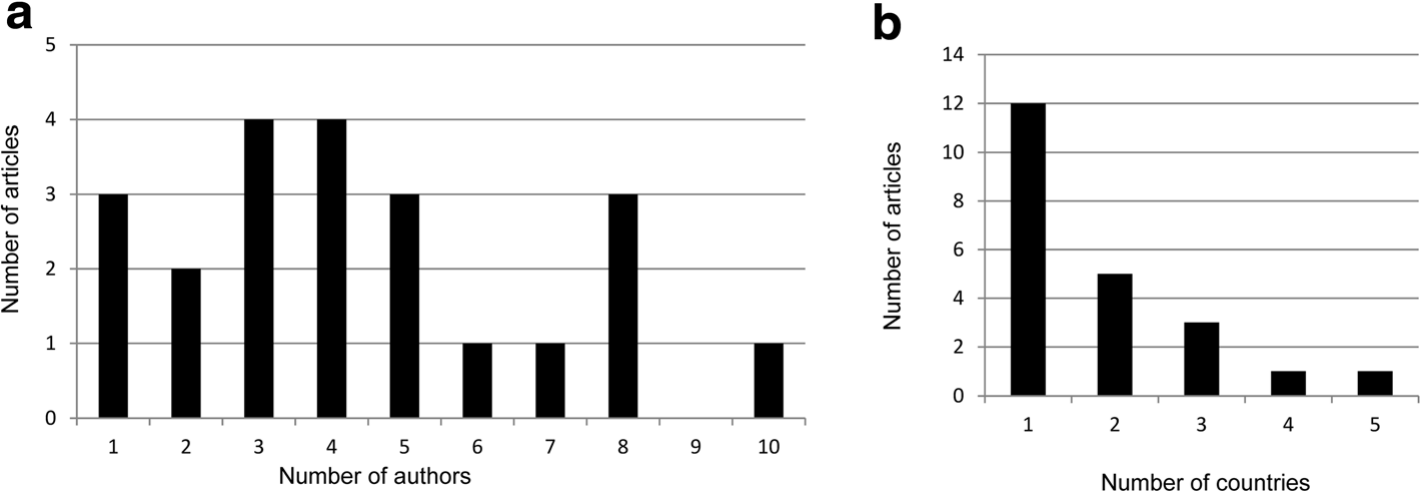
Histogram showing the number of authors per article (**a**) and the number of countries the contributing authors are affiliated with (**b**) in a collection of 22 contributions forming a thematic issue of *Infectious Diseases of Poverty* pertaining to surveillance-response systems that might lead to elimination of tropical diseases

As shown in Fig. 2a, there was a spectrum of author constellations ranging from three single-author contributions [12, 16, 21] to collaborative pieces co-authored by up to 10 researchers [11]. More than three-quarters (77.3 %) of the articles were written by at least three authors, while the median number of authors per article is four. Among the 19 multi-authored contributions, nine papers have been written by authors affiliated with a single country, while the remaining 10 contributions are composed of authors affiliated with different countries. One of the articles has contributing authors affiliated with four different countries [15] and another one with five different countries or regions [17] (Fig. 2b). Hence, the research pursued in this collection represents work of considerable collaborative nature.

A sum-up with respect to authorship reveals a total of 97 contributing scientists. Most of them contributed to a single piece (*n* = 90, 92.8 %), while the remaining seven authors were engaged with two or more articles, up to a maximum of four contributions [8, 11, 17, 26].

**Fig. 3:**
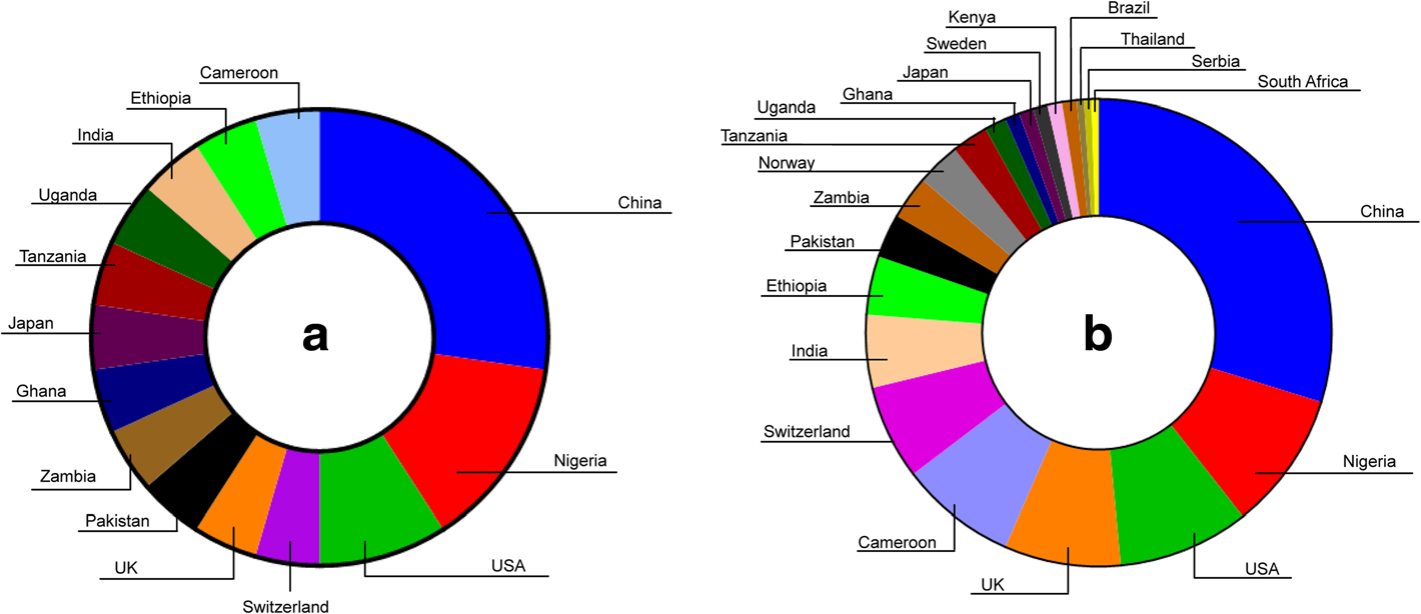
Provenance of 22 contributions (**a**) and the 97 author-contributions (**b**), stratified by authors’ country affiliations, in a collection of *Infectious Diseases of Poverty* pertaining to surveillance-response systems that might lead to elimination of tropical diseases

The provenance of the 22 contributions is depicted in Fig. 3a, with the provenance of the 97 author-contributions displayed in Fig. 3b. In the latter analysis, each contribution is given the same weight, and hence, the 97 author-contributions account for 100 %. Authors with affiliations in two different countries (e.g. P.R. China and South Africa) were accounted for as half for each country. We found that authors affiliated with institutions in P.R. China contributed the highest number of articles (*n* = 31.8, 32.8 %), followed by Nigeria (*n* = 9.0, 9.3 %), the United States of America (USA; *n* = 8.0, 8.2 %), Cameroon (*n* = 8.0, 8.2 %) and the United Kingdom (UK; *n* = 7.3, 7.5 %). Author-contributions from Asia amounted to 41.3 % (40.1/97) followed by author-contributions from Africa 31.7 % (30.8/97), whilst author-contributions from Europe, North America and South America represent 17.6 %, 7.4 % and 1.0 %, respectively.

In the article-specific analysis, each of the 22 papers was given the same weight. It follows that articles written by a single or only few authors contributed considerably more weight to a given country than articles prepared by several authors from various countries. Taking this weighting into account, P.R. China remains the dominant player (32.8 %), followed by Nigeria (9.3 %) and the USA (8.2 %).

## Highlights

### Overview

In their opening overview, Zhou and colleagues [11] provide a comprehensive review on the framework of elimination of tropical diseases by surveillance-response systems. Current strategies and the WHO’s roadmap for elimination of neglected tropical diseases (NTDs) [31] are reviewed. A priority research agenda within the “One World – One Health” framework for global health has been developed, including (i) the establishment of a platform for resource-sharing and effective surveillance-response systems for Asia Pacific and Africa with an initial focus on efforts aimed at the elimination of lymphatic filariasis, malaria and schistosomiasis; (ii) development of new strategies, tools and approaches, such as improved diagnostics and antimalarial therapy; (iii) rigorous validation of surveillance-response systems; and (iv) designing pilot studies for the transfer of Chinese experience of successful surveillance-response systems to endemic countries with limited resources. To emphasise the objectives of this thematic issue based on a “One World – One Health” paradigm, an editorial summarises the outcomes of the First *Forum on Surveillance Response Systems Leading to Tropical Diseases Elimination*, held in Shanghai in June 2012, including identified research priorities [3, 8, 32].

### Data handling and databases

Modelling based on a minimum essential database had been proposed in the First *Forum on Surveillance Response System Leading to Tropical Disease Elimination* and was reiterated in the second forum, held in June 2014, again in Shanghai. A key outcome of the two conferences was the writing-up of the 22 articles featured here. This body of work emphasises the essential role that a minimum essential database can play for effective surveillance and responses that are readily tailored for specific settings.

Data on treatments, co-infections and prevalence of a large variety of tropical diseases are provided in this collection. One scoping review put forth by Liu et al. [12] emphasises that chloroquine-resistant *Plasmodium falciparum* malaria exists in eight provinces/autonomous regions of P.R. China. This type of resistance is particularly widespread in the provinces of Hainan and Yunnan. The issue also covers formulations and recommendations of therapeutic regimens for antimalarial drug use in P.R. China for artemisinin/pyronaridine compounds to promote the rational use of antimalarials and strengthen malaria control/elimination efforts [12].

Contemporary data on immunological, haematological and viral responses and predictors of reported therapeutic failure after initiation of free antiretroviral treatment in Cameroon were collected to evaluate existing treatment-monitoring algorithms and to complement efforts to scale-up and improve the management of HIV/AIDS [20].

Since the early 1990s, it has been suggested that HIV and *S. mansoni* may interact and potentiate the effects of each other within the co-infected human host. A previous collection of *Infectious Diseases of Poverty* included two articles on the topic of co-infections with one article pertaining to HIV-1/AIDS and *S. mansoni* that are both widespread in sub-Saharan Africa [15], and the second elucidating on the impact of HIV on TB [30]. The implications of co-infections, both for TB and HIV/AIDS control, are of considerable public health relevance in Ghana, as almost a quarter (23 %) of all TB cases were HIV positive according to a report from 2010. The integration of TB and HIV services has therefore emerged as an essential component of the national response to TB and HIV co-infections in this country [30].

Dual burden of disease and other special physical conditions are also covered in this collection , e.g. the report by Amare and colleagues who observed a 6.2 % prevalence of smear-positive pulmonary TB (PTB) in diabetic patients, which turned out to be several-fold higher compared with the general population (0.39 %). Patients with a previous history of contact with TB patients, as well as those with prolonged diabetes, were found to be particularly prone to have PTB, making it necessary to screen diabetic patients for PTB infection at follow-up medical visits [27].

Agomo et al. observed that acquired partial immunity against malaria during pregnancy is a key factor in keeping this infection at an asymptomatic level. Depending on the malaria endemicity in a given setting, it is expected that up to 50 % of pregnant women may have parasitaemia, especially in the placenta, although they might not be aware of this [24].

### Modelling approaches

A mathematical model for the prediction of risk of infection through needle/syringe-sharing during mass vaccination, including a formula for its calculation, has been developed by Okamoto [21]. Another two research articles with an emphasis on modelling, put forward by Wiwanitkit et al. [17] and Shi et al. [26], discuss the potential risks of H7N9 infection by spatio-temporal characterisation of bird migration and poultry distribution, and estimation of the basic reproduction number for single-strain dengue fever epidemics. The epidemic of H7N9 avian influenza A in the eastern part of P.R. China in early 2013 caused much attention from researchers as well as public health workers, and modelling of its transmission risk resulted in guidelines for active surveillance and control of human influenza infections through intensive intervention in poultry markets. In their article pertaining to dengue transmission by estimating the basic reproduction number for single-strain dengue fever epidemics, researchers from Pakistan did a retrospective analysis of the 2011 epidemic in that region [19].

### Application of more sensitive diagnostics

Use of new technologies supported by mobile or other improved electronic-based approaches and more sensitive diagnostic tools, necessary for settings chosen for elimination, were identified as a key research priority in the First *Forum on Surveillance Response System Leading to Tropical Disease Elimination* in June 2012 [8]. No-cost social media (Weibo) [10] and mobile phones [25] were employed in two studies to evaluate reactions of the general public with regard to infectious diseases [10, 25].

Applications of new molecular diagnostic tests characterised by a high sensitivity, such as the loop-mediated isothermal amplification (LAMP), for real-time diagnosis of malaria infections in a rural or urban high/low transmission setting are recommended in the report of the *Second Forum on Surveillance Response System Leading to Tropical Disease Elimination* of June 2014 (http://srs.ipd.org.cn:8080/srs/node/9). Meanwhile, the serological markers for monitoring of *Plasmodium vivax* transmission in the elimination stage were identified by means of a high throughput analysis [33] to develop more sensitive and convenient diagnostics.

### Drug resistance and prevention

To promote the rational use of antimalarial drugs and strengthen malaria control and elimination, established drug resistance and surveillance of antimalarial drug resistance in P.R. China in the 1980s and 1990s have now been documented, including the formulation of principles and recommendations of therapeutic regimens for the use of antimalarial drugs (artemisinin/pyronaridine compounds) [12]. To identify an antifilarial drug target, *in vitro* ‘gene silencing’ of independent phosphoglyceratemutase (iPGM) in the filarial parasite *B. malayi* was performed by Singh et al., whose work suggests that iPGM is essential for both larval and adult stages of *B. malayi* [28].

Dr. William H. Stewart, United States Surgeon General from 1965 to 1969, has been maligned for the statement: “It is time to close the book on infectious diseases, and declare the war against pestilence won”. It must be noted, however, that recent investigations fail to find any evidence for him ever having said this [29]. More importantly, in retrospect, emerging new infections and the ever-increasing problems of resistance against available antibiotics clearly shows that now is not the time to close the book on infectious dieases.

### Socioeconomics and infectious diseases

Among the 22 articles pertaining to surveillance-response systems for elimination of tropical diseases, four are associated with socioeconomic factors in measuring health determinants. Data from social media (Weibo) and mobile phones were analysed in two studies to better understand reactions of the general public to infectious diseases. One study, carried out by Fung et al. [10], revealed that Weibo (microblogs freely published through the Internet), proved useful to measure the Chinese people’s reactions with respect to two different outbreaks, namely the 2012 MERS-CoV outbreak [34] and the 2013 outbreak of H7N9 avian influenza A [35, 36]. Hence, the results suggest that Weibo could be a useful measure to raise public awareness by having health authorities release disease outbreak information *via* Weibo.

Kliner et al. [25] used a no-cost mobile phone agreement to send outpatient reminders for HIV testing and counselling in a pilot study in rural Swaziland, and found that mobile technology leading towards the concept of mobile health (mHealth) can be useful in many ways, initially by improving attendance at outpatient service clinics. Work stemming from Nigeria assessed household payments for TB with regard to incidence, determinants and policy implications for universal health coverage. The results suggest that current health cost-lowering strategies in Nigeria are not sufficient to prevent households from incurring catastrophic out-of-pocket payments for TB care [23]. To assess resources for the use of community-directed interventions (CDI), another study from Nigeria investigated delivery of health interventions in poorly-served urban communities in Ibadan. The research support the feasibility of using the CDI process in delivering health interventions in urban, poor environments and shows that resources for the strategy were potentially abound in the communities.

## Visibility

### Scope and transdisciplinary nature of the work

Five articles reported on the estimation of disease burdens by measuring the impact of diseases at the local, national and global levels. With regard to rabies, one study collected all published articles and official documents on the disease in P.R. China in order to examine challenges to its elimination [13], while another discussed a global approach to rabies elimination emphasising the efforts needed to strengthen research and control activities, particularly in low- and middle-income countries [16].

Qian et al. [18] provided a concerted opinion on the tackling of clonorchiasis in P.R. China, while a review commented on the epidemiological status and characteristics of the disease at the global level and the aetiological relationship between cholangiocarcinoma and this infection [37]. The significance for clonorchiasis control and research priorities in P.R. China have been emphasised according to the pattern of recent epidemics and trend analyses in two papers [38, 39].

TB at the wildlife-livestock-human interface in Zambia was reviewed by Malama et al. [14], who called attention to the complex connection between the burden of animal *Mycobacterium tuberculosis* infections and its public health implications. The review pertaining to the epidemiology and interactions of HIV-1 and *S. mansoni* in sub-Saharan Africa indicates that *S. mansoni* infections can influence the replication of HIV-1 and deworming of HIV-positive individuals living in endemic areas, as it may impact on HIV-1 viral loads and CD4^+^T lymphocyte counts [15].

Four research articles discuss the role of governments in disease endemic-countries and encourage cooperation in the field of zoonotic diseases [13, 15, 18, 26]. Another investigation highlights the collaboration between various governmental ministries as well as among medical and veterinary doctors by employing the “One World – One Health” concept [14].

### Innovative technology

Surveillance-response approaches regarding tropical diseases in resource-constrained settings are increasingly applied in endemic countries with ongoing control and elimination programmes. Articles targeting such issues, taking into account environmental changes, social determinants and health systems considerations are in the limelight of the collection reviewed here. Indeed, among the 22 contributions, three have been viewed over 10,000 times within 4 months, 15 months and 22 months, respectively, after their publication [10, 24, 26]. The development of an innovative modelling approach entitled “Inferring the potential risks of H7N9 infection by spatiotemporally characterising bird migration and poultry distribution in eastern China”, had the most views (14,904; accessed on 31 August 2015). Research on the social factors affecting the spread of infectious diseases entitled “Chinese social media reaction to the MERS-CoV and avian influenza A (H7N9) outbreaks” followed with 11,759 views.

In summary, it is fair to say that the importance of surveillance-response systems, representing the final crucial steps towards achieving effective control and elimination of communicable diseases, caused more attention than any other papers in this thematic issue of *Infectious Diseases of Poverty*.

In our analysis on the breadth and depth of the 22 contributions, we found that Chinese authors are becoming increasingly active with respect to control and elimination of tropical diseases. In concurrence with patterns recently published by Utzinger et al. [40] and Bergquist et al. [31, 40], the application of surveillance-response systems have developed into a necessary and sustainable part of the Chinese national disease control and elimination programmes [40–44].

## Conclusions

Among the original research articles providing the core of this collection, seven research priorities emerged: (i) dynamic mapping of the transmission potential of various diseases; (ii) providing tools in near real-time capture of population dynamics; (iii) modelling based on minimum, essential databases; (iv) implementation of mHealth; (v) development of sensitive diagnostics; (vi) design of response packages tailored to different transmission settings and levels; and (vii) validation of surveillance and responses packages.

Through development and application of surveillance-response systems as part of the national diseases control and elimination programmes [40–44], P.R. China is becoming an important player in the area of the control and elimination of tropical diseases. It is in this connection essential that P.R. China, as one of the major contributors to the expansion of global health programmes, translates its experience into research and control of infectious diseases of poverty in places where these diseases are still widespread, including parts of P.R. China itself.

## Additional files

Additional file 1: Multilingual abstracts in the six official working languages of the United Nations. (PDF 253 kb) Additional file 2: List of the 22 articles in the thematic issue of *Infectious Diseases of Poverty* pertaining to surveillance-response systems that might lead to elimination of tropical diseases. (XLSX 158 kb)
