# Supplementary material for: Surveillance and response systems for elimination of tropical diseases: summary of a thematic series in Infectious Diseases of Poverty
Source: Infect Dis Poverty. 2016 May 14;5:49. doi: 10.1186/s40249-016-0144-7 (PMC4868018; doi:10.1186/s40249-016-0144-7)
Supplement: Additional file 1: — Multilingual abstracts in the six official working languages of the United Nations. (PDF 253 kb) [file 40249_2016_144_MOESM1_ESM.pdf]

Translation of the abstract into the six official working languages of the United Nations

نظم المراقبة والاستجابة للقضاء على الأمراض المدارية: موجز لمجموعة مواضيعية في "الأمراض المعدية مصدرها الفقر "

تشو شيا، بيلينج ياب، مارسيل تانر، روبرت بيرجكويست، يورغ أوتزينجير، تشو شياو نونغ

ملخص

الدورية المهنية باسم "الأمراض المعدية مصدرها الفقر" هي عبارة عن منتدى جديد للدراسة و النشر بأسلوب مفتوح للجميع للعلوم خارج حدود التخصصات التقليدية. الموضوع المقدم هنا يستعرض مجموعة مواضيعية في نظم المراقبة -الاستجابة للقضاء على أمراض المناطق المدارية. عموما، هناك 22 من المساهمات من المقالات تستعرض مجموعة واسعة من الأمراض – منها أمراض متفرعات الخصية وحمى الضنك، والتهاب الكبد، وفيروس نقص المناعة البشرية/متلازمة نقص المناعة المكتسب (الإيدز)، إنفلونزا الطيورH7N9 ، داء الخيطيات اللمفاوية، الملاريا، أمراض الالتهاب الرئوي لمنطقة الشرق الأوسط، داء الكلب، البلهارسيا والدرن (السل). وهناك خمس مراجعات وتعليق واحد ورسالة إلى المحرر ومقال للرأي ومقال افتتاحي تتعلق بموضوع "القضاء على أمراض المناطق المدارية من خلال جهود المراقبة والاستجابة".

المواد ال 13 المتبقية هي المساهمات الأصلية تغطي ما يلي: (أولا) مقاومة الدواء؛ (ثانيا) الابتكار والتحقق في مجال النمذجة الرياضية؛ (ثالثا) القضاء على الأمراض المعدية؛ و (رابعا) التقارير التحذيرية الصادرة عن السلطات الصحية الوطنية على شبكات التواصل الاجتماعي حول تفشي المرض. ويكشف تحليل للارتباطات المهنية للمؤلفين أن العلماء من جمهورية الصين الشعبية لهم حضور بارز. والتفسيرات المحتملة لذلك تشمل الواقع أن المؤتمرات الدولية للسنوات 2012 و2014 المرتبطة بآليات المراقبة والاستجابة قد تم استضافتها من قبل المعهد الوطني للأمراض الطفيلية في شانغهاي، إلى جانب الاهتمام المتزايد لجمهورية الصين الشعبية بموضوع السيطرة على الأمراض المعدية. وفي غضون 4 إلى 22 شهرا من النشر، جرى الاطلاع على ثلاثة من إجمالي 22 من المساهمات لأكثر من 10000 مرة لكل منها. وبفضل بذل الجهود المتواصلة مع التركيز على المعلومات ذات الصلة واتباع استراتيجية للسيطرة والقضاء على الأمراض المعدية، فقد أصبحي هذه الدورية "الأمراض المعدية مصدرها الفقر" مرجعاً يومياً رائداً في مجال نظم المراقبة والاستجابة في ميدان الأمراض المعدية وما بعدها

Translated from English version into Arabic by Lamya, through

TRANSLATORS
WITHOUT BORDERS

消除热带病的监测和应对体系：《贫穷相关传染病》杂志专题系列文章小结

周霞, Peiling Yap, Marcel Tanner, Robert Bergquist, Jürg Utzinger, 周晓农

摘要:

同行评议开放性期刊《贫困相关传染病》杂志在传统学科之外为学科交流提供了一个新的交流与互动平台。本文回顾了消除热带病的监测与反应体系相关的专题系列文章。该系列共发表相关论文22篇广泛涵盖了一系列疾病，包括即肝吸虫病、登革热、肝炎、人类免疫缺陷病毒/获得性免疫缺陷综合症（HIV/AIDS）、H7N9禽流感、淋巴丝虫病、疟疾、中东呼吸道综合征（MERS）、狂犬病、血吸虫病和结核病（TB）等。其中有5篇综述，1篇评论、1篇读者给编辑的来信、1篇观点论述和1篇编辑撰稿题为“热带病的消除，通过监测和应对体系”的评论。其余13文章均为原创性著作主要包括（一）耐药性；（二）数学建模领域的创新和应用；（三）消除传染性疾病；（四）社交媒体的报道的国家卫生部门发布疫情通报四个方面。2012年和2014年的两次国际传染病监测与反应体系会议都在上海由中国疾病预防控制中心寄生虫病研究所（NIPD）承办，加上中国对关于传染病的控制日益重视，顾从作者隶属分析表明，来自中国的科学家贡献最突出。在发表后4~22个月内，共发表的22篇文章中的三篇文章访问量达每10 000次以上。随着集中在对控制和消除传染病的相关信息和战略不断探索，《贫困相关传染病》杂志已成为传染病及以后的监测和应对系统领域的领先期刊。

Translated from English version into Chinese by Zhou Xia

Systèmes de surveillance et de réponse pour l’élimination des maladies tropicales : résumé d’une série thématique dans

Infectious Diseases of Poverty

Xia Zhou, Peiling Yap, Marcel Tanner, Robert Bergquist, Jürg Utzinger, Xiao-Nong Zhou

Résumé

La revue à comité de lecture *Infectious Diseases of Poverty* offre une plate-forme pour aborder la science par-delà des frontières traditionnelles des disciplines et la diffuser sous un format ouvert. Le présent article passe en revue une série thématique consacrée aux systèmes de surveillance et de réponse pour l'élimination des maladies tropicales. Celle-ci regroupe 22 contributions, couvrant un large éventail de maladies : clonorchiase, dengue, hépatite, virus de l'immunodéficience humaine/syndrome d'immunodéficience acquise (VIH/SIDA), grippe aviaire H7N9, filariose lymphatique, paludisme, syndrome respiratoire du Moyen-Orient (MERS), rage, schistosomiase et tuberculose. Ces publications comprennent cinq études de portée, un commentaire, une lettre à la rédaction, une chronique d'opinion et un éditorial sur le thème « Élimination des maladies tropicales par la surveillance et la réponse ». Les 13 articles restants sont des contributions originales, portant principalement sur (i) la résistance aux médicaments, (ii) l'innovation et la validation en modélisation mathématique, (iii) l'élimination des maladies infectieuses et (iv) la transmission dans les réseaux sociaux des notifications d'épidémies publiées par les autorités nationales de la santé. L'analyse des affiliations des auteurs montre que les scientifiques de la République populaire de Chine (RPC) sont fortement représentés. Cela peut s'expliquer par le fait que les conférences internationales de 2012 et 2014 sur les mécanismes de surveillance et de réponse ont été accueillies par l'Institut national des Maladies parasitaires (NIPD) à Shanghai, et aussi par l'importance croissante de la Chine dans la lutte contre les maladies infectieuses. Dans un délai de 4 à 22 mois après leur publication, 3 des 22 communications ont été lues chacune plus de 10 000 fois. En concentrant ses efforts sur les informations pertinentes et stratégiques dans la lutte contre les maladies infectieuses et leur élimination, *Infectious Diseases of Poverty* est devenue une revue de premier plan dans le domaine de la surveillance et de la réponse aux maladies infectieuses et au-delà.

Translated from English version into French by Suzanne Assenat, through

TRANSLATORS
WITHOUT BORDERS

Системы наблюдения и реагирования для устранения тропических болезней: Краткое изложение тематической серии в области *Инфекционных Болезней Бедности*

Ся Чжоу, Пейлинг Яп, Марсель Таннер, Роберт Бергкуист, Юрг Ютзингер, Сяо-Нонг Чжоу

Аннотация

В рецензируемом журнале инфекционных болезней бедности предоставляется новая платформа для взаимодействия с, и распространение в формате открытого доступа, науки за пределами традиционных дисциплинарных границ. В настоящая часть рассматривает тематической серии по системам наблюдения и реагирования для устранения тропических болезней. В целом, 22 вкладов, охватывающие широкий спектр заболеваний являются особым - т.е. клонорхоз, денге, гепатит, вирус иммунодефицита человека / синдром приобретенного иммунодефицита (ВИЧ / СПИД), H7N9 птичий грипп, лимфатический филяриатоз, малярия, Ближневосточный респираторный синдром (БВРС), бешенство, шистосомоз и туберкулез (ТБ). Есть пять охватывающий обзор, комментарий, письмо к редактору, статья-мнение и редакционная статья относящейся к теме «ликвидации тропических болезней путем эпиднадзора и ответных мер". Остальные 13 статей являются ценными вкладами в основном охватывает (I) устойчивости к лекарственным средствам; (II) инновации и проверки в области математического моделирования; (III) ликвидация инфекционных заболеваний; и (IV) отчеты социальных медиа об уведомления вспышек

болезни, выпущенных национальными органами здравоохранения. Анализ сведении о принадлежности автора показывает, что ученые из Китайской Народной Республики Китай (Китайская Народная Республика) заметно представлены. Возможные объяснения включают тот факт, что в 2012 и 2014 годах международные конференции, имеющие отношение к механизмам наблюдения и реагирования оба, были организовано Национальным институтом паразитарных заболеваний (НИПЗ) в Шанхае, наряду с растущей важности Китайской Народной Республики в отношении борьбы с инфекционными заболеваниями. В течение 4-х до 22 месяцев после опубликования, три из 22 вкладов были просмотрены более чем в 10000 раз каждый. С настойчивыми усилиями, сосредоточив внимание на актуальной и стратегической информации в направлении контроля и ликвидации инфекционных заболеваний, инфекционных болезней бедности стала ведущим журналом в области системы эпиднадзора и ответных мер при инфекционных заболеваниях и за ее пределами.

Translated from English version into Russian by Turdimurot Rakhmonov, through

TRANSLATORS
WITHOUT BORDERS

Sistemas de vigilancia y respuesta para la eliminación de enfermedades tropicales: resumen de una serie temática en la publicación académica *Infectious Diseases of Poverty*

Xia Zhou, Peiling Yap, Marcel Tanner, Robert Bergquist, Jürg Utzinger, Xiao-Nong Zhou

Resumen

La publicación académica *Infectious Diseases of Poverty* ofrece una nueva plataforma de interacción y divulgación, en un formato de acceso abierto, de la ciencia más allá de sus disciplinas tradicionales. El presente artículo revisa una serie temática de sistemas de vigilancia-respuesta para la eliminación de enfermedades tropicales. En total se incluyen 22 colaboraciones que cubren una amplia variedad de enfermedades, p.ej., clonorquiasis, dengue, hepatitis, virus de inmunodeficiencia humana/síndrome de inmunodeficiencia adquirida (HIV/SIDA), gripe aviar H7N9, filariasis linfática, malaria, síndrome respiratorio de Oriente Medio (MERS), rabias, esquistosomiasis y tuberculosis (TB). Hay cinco revisiones, un comentario, una carta al director, un artículo de opinión y una editorial sobre el tema “Eliminación de enfermedades tropicales mediante vigilancia y respuesta”. Los 13 artículos restantes son colaboraciones que cubren principalmente (i) resistencia a los fármacos; (ii) innovación y validación en el campo del modelado matemático; (iii) eliminación de enfermedades infecciosas; y (iv) informes de medios sociales sobre brotes de enfermedades emitidos por las autoridades sanitarias nacionales. El análisis del origen de los autores indica que los científicos de la República Popular China están ampliamente representados. Entre las posibles razones que lo explican están el hecho de que en 2012 y 2014 se celebraron conferencias internacionales correspondientes a mecanismos de vigilancia-respuesta en el Instituto Nacional de Enfermedades Parasitarias de Shanghai, junto con la creciente importancia de China relacionada con el control de enfermedades infecciosas. Entre 4 y 22 meses de publicación se vieron tres de las 22 colaboraciones más de 10.000 veces cada una. Con esfuerzos sostenidos centrados en información relevante y estratégica para el control y la eliminación de enfermedades infecciosas, *Infectious Diseases of Poverty* se ha convertido en una publicación de referencia en el campo de los sistemas de vigilancia y respuesta en enfermedades infecciosas y de otro tipo.

Translated from English version into Spanish by SergioLorenzi, through

TRANSLATORS
WITHOUT BORDERS
